# Editorial: From Biology to Clinical Management: An Update on Aortic Valve Disease

**DOI:** 10.3389/fcvm.2019.00004

**Published:** 2019-01-23

**Authors:** Cécile Oury, Alain Nchimi, Patrizio Lancellotti

**Affiliations:** ^1^Department of Cardiology, GIGA Cardiovascular Sciences, Heart Valve Clinic, CHU Sart Tilman, University of Liège Hospital, Liège, Belgium; ^2^Gruppo Villa Maria Care and Research, Anthea Hospital, Bari, Italy

**Keywords:** aortic valve (AV), aortic valve replacement (AVR), aortic valve calcification, aortic stenosis (AS), TAVI—transcatheter aortic valve implantation

Calcific aortic stenosis (AS) is the most frequent valvular heart disease in Western countries, affecting up to 13% of individuals over 75 years (1, 2). The disease is associated with considerable morbidity and mortality. Major risk factors include older age, congenital anomalies of the aortic valve (bicuspid valve), male gender, hypertension, dyslipidaemia, smoking, and diabetes (3).

The disease is characterized by fibro-calcification of aortic valve cusps and concomitant left ventricular (LV) remodeling due to chronic pressure overload, which can evolve into overt heart failure. AS progresses very slowly until the onset of symptoms (angina, dyspnae, syncope). A large majority of patients remain asymptomatic for a long period, though at increased risk for untoward events (death, heart failure, symptomatic deterioration, LV dysfunction). Development of symptoms is a class I indication for aortic valve replacement (AVR). Today, about 300,000 AVR are performed annually worldwide, either via surgery (SAVR) or transcatheter implantation (TAVI). AVR is indeed the only treatment shown to improve survival. There is no pharmacological treatment to prevent or slow disease progression.

The present research topic provides a comprehensive overview of AS clinical management with a special focus on valve prostheses, imaging and blood biomarkers as well as on recent advances on pathophysiology and valve biology.

Regarding valve prostheses, the ideal prosthesis either mechanical or biological still do not exist. Current prosthesis can cause complications, which necessitate reoperation or lead to death in 50–60% of patients within 10 years post-implantation. In this research topic, Musumeci et al. reviewed the different types of currently available prosthetic aortic valves and their limitations. It appears that thrombosis, infection, bioprosthesis calcification, and degeneration remain major issues, which could be addressed through innovative new generation prostheses.

Rachwan et al. report on a patient who presented with a thrombus on a bicuspid aortic valve in the setting of antiphospholipid syndrome (APLS). APLS is a systemic autoimmune disease defined by thrombotic events in patients persistently positive for antiphospholipid antibodies (aPL). In this case report, 4-months moderate-intensity anticoagulation efficiently eliminated the aortic valve thrombus. However, due to the rarity of this condition, whether conservative anticoagulation or AVR should be recommended remains to be determined. More generally, there is currently no clear recommendation on the choice of antithrombotic regimen for AS patients (4, 5).

Another major challenge in the clinical management of AS is deciding on the correct timing of AVR (6).

Regarding clinical imaging, echocardiography is central to the diagnosis and risk stratification of patients with aortic stenosis and regurgitation. However, the technique has certain limitations, and aortic valve imaging may benefit from alternative and complimentary multimodality imaging. In the present topic, Nchimi et al. performed a systematic review and meta-analysis in order to evaluate the role of imaging biomarkers in predicting AS progression to clinical symptoms and mortality. Eight studies regrouping 1,639 patients were included in the analysis. This study showed significant associations of computed tomography aortic valve calcification (AVC) and myocardial fibrosis, measured by cardiac magnetic resonance (CMR), with clinical outcomes. The findings on AVC are in line with a recent study showing that sex-specific AVC thresholds accurately identify severe AS and predicts AVR and death (7). Late enhancement gadolinium fibrosis was significantly associated with cardiac mortality, which is in agreement with another recent meta-analysis indicating that LV fibrosis can also have prognostic value after AVR (8). Hence, the prognostic efficacy of these imaging biomarkers for patient management as compared to the current approach that relies mainly on clinical performance need to be tested in large randomized studies.

In addition to clinical imaging, several studies strongly suggest that circulating biomarkers could help for AS patient risk stratification (9). In this research topic, Oury et al. reviewed the role of circulating biomarkers in patients undergoing TAVI. Despite the fact that TAVI offers a marked change in life expectancy and quality of life of high-risk elderly patients, (10) early and late mortality after TAVI still remains relatively high (11–13). Studies indicate that implementing biomarkers of myocardial injury, cardiac mechanical stretch, inflammation, and of hemostasis imbalance in clinical practice might help reducing TAVI-associated complications and mortality. However, the role of these biomarkers has yet to be confirmed in large randomized studies.

Nevertheless, the identification of novel biomarkers will necessitate a better understanding of aortic valve biology and mechanisms of disease. The review by Hulin et al. draws a summary of current knowledge on pathogenic pathways and their potential role as novel therapeutic targets. Heart valve homeostasis is tightly controlled by valve interstitial cells (VICs) embedded in extracellular matrix, valve endothelial cells (VECs) covering the leaflet, and circulating and resident immune cells. AS is now considered as an active multi-step process. Early steps of lesion development would occur through accumulation of lipids and free cholesterol within the fibrosa, followed by infiltration of inflammatory cells, e.g., macrophages and T lymphocytes. VICs then enter an osteogenic program, initiating calcium nodule formation, and valve calcification (2). All these events likely involve mechanical stress and strain, and a major role for valve lining endothelial cells. However, how these complex cellular interplay contributes to AS remains unknown. Furthermore, thorough knowledge of the heterogeneity and function of valve cell subtypes, over the course of the disease, may provide useful informations to develop targeting strategies of diseased cells. In this sense, transcriptional profiling studies during valve development could help to better define valve tissue composition and homeostatic biological pathways. In this research topic, Nordquist et al. compared mRNA expression in postnatal and adult valve tissues. This study nicely unveiled multiple conceivable processes that contribute to postnatal valve maturation and maintenance that may pave the way for elucidating mechanisms underlying valve defects.

Thus, this research topic highlights important unmet medical needs in AS. More basic and translational research is definitely required to clarify disease mechanisms, uncover new multi-biomarker-based diagnostic and prognostic tools, and develop more biocompatible and durable prostheses with the goal of improving patient outcome.

## Author Contributions

All authors listed have made a substantial, direct and intellectual contribution to the work, and approved it for publication.

### Conflict of Interest Statement

The authors declare that the research was conducted in the absence of any commercial or financial relationships that could be construed as a potential conflict of interest.
